# A Quality Improvement Project to Investigate How Addenbrooke's Cognitive Examination-III (ACE-III) Training Improves the Accuracy of ACE-III Scoring in an Older Adult Community Mental Health Team

**DOI:** 10.1192/bjo.2024.448

**Published:** 2024-08-01

**Authors:** Zara Tyson

**Affiliations:** Devon Partnership Trust, Devon, United Kingdom

## Abstract

**Aims:**

Aims – An Audit in the Older Adult Community Mental Health Team identified that there were inaccuracies in the Addenbrooke's Cognitive Examination-III (ACE-III) scoring used to help diagnose dementia. The aim of this Quality Improvement Project was to determine if ACE-III training delivered by a neuropsychologist would improve the accuracy and reliability of ACE-III scores used by the team to help diagnose dementia.

**Methods:**

ACE-III surveys completed over a 6 month period were analysed to determine if they followed the ACE-III scoring guidelines provided by the ACE-III Administration and Scoring Guide (2012). ACE-III surveys were completed by different members of the multidisciplinary team. Following identification of inaccuracies and inconsistencies in scoring we delivered ACE-III training via a neuropsychologist to determine if this would improve ACE-III scoring (as per the ACE-III Administration and Scoring Guide) in the following 6 month period after the training was received.

**Results:**

Following ACE-III training delivered by a neuropsychologist in how to complete the ACE-III survey, surveys were analysed using the Administration and Scoring Guide (2012). ACE-III scores were more accurate in the 6 months following the ACE-III training delivered by a neuropsychologist to the team.

**Conclusion:**

ACE-III training improved the accuracy of ACE-III scores in the multidisciplinary CMHT. This finding would advocate for ACE-III training to become part of our roles within Older Adult Psychiatry in order to improve service delivery to the patient.

